# Role of leukocytes, gender, and symptom domains in the influence of depression on hospitalization and mortality risk: Findings from the Moli-sani study

**DOI:** 10.3389/fpsyt.2022.959171

**Published:** 2022-10-13

**Authors:** Alessandro Gialluisi, Francesca Bracone, Simona Costanzo, Federica Santonastaso, Augusto Di Castelnuovo, Sabatino Orlandi, Sara Magnacca, Amalia De Curtis, Chiara Cerletti, Maria Benedetta Donati, Giovanni de Gaetano, Licia Iacoviello

**Affiliations:** ^1^EPIMED Research Center, Department of Medicine and Surgery, University of Insubria, Varese, Italy; ^2^Department of Epidemiology and Prevention, IRCCS NEUROMED, Pozzilli, Italy; ^3^Mediterranea Cardiocentro, Napoli, Italy

**Keywords:** depression, mortality, hospitalizations, inflammation, granulocytes, lymphocytes, cardiovascular disease, C-reactive protein

## Abstract

**Background:**

Major depressive disorder is a mental illness associated with chronic conditions like cardiovascular disease (CVD). Circulating inflammation has been proposed as a potential mechanism underlying this link, although the role of specific biomarkers, gender, and symptom domains is not well elucidated.

**Methods:**

We performed multivariable Cox regressions of first hospitalization/all-cause mortality and CVD, ischemic heart (IHD), and cerebrovascular disease (CeVD) causes vs. depression severity in an Italian population cohort (*N* = 13,191; age ≥ 35 years; 49.3% men; 4,856 hospitalizations and 471 deaths, median follow-up 7.28 and 8.24 years, respectively). In models adjusted for age, sex, and socioeconomic status, we estimated the proportion of association explained by C-reactive protein (CRP), platelet count, granulocyte-to-lymphocyte ratio (GLR), and white blood cell count (WBC). Gender-by-depression interaction and gender-stratified analyses were performed. Associations of polychoric factors tagging somatic and cognitive symptoms with incident clinical risks were also tested, as well as the proportion explained by a composite index of circulating inflammation (INFLA score).

**Results:**

Significant proportions of the influence of depression on clinical risks were explained by CRP (4.8% on IHD hospitalizations), GLR (11% on all-cause mortality), and WBC (24% on IHD/CeVD hospitalizations). Gender-by-depression interaction was significantly associated only with all-cause mortality (*p* = 0.03), with moderate depression showing a + 60% increased risk in women, but not in men. Stable associations of somatic, but not of cognitive, symptoms with increased hospitalization risk were observed (+ 16% for all causes, + 14% for CVD causes), with INFLA score explaining small but significant proportions of these associations (2.5% for all causes, 8.6% for IHD causes).

**Conclusions:**

These findings highlight the importance of cellular components of inflammation, gender, and somatic depressive symptoms in the link between depression and clinical (especially CVD) risks, pointing to the existence of additional pathways through which depression may play a detrimental effect on the cardiovascular system.

## Introduction

Depression is a common mental illness with a lifelong prevalence of 4.4% worldwide (1). There are several subtypes, with symptoms ranging in terms of their severity (from mild to severe) and duration (from months to years) (2). Among these, major depressive disorder (MDD) represents the most severe form (3, 4). MDD shows diverse signs and symptoms, like low mood, loss of interest or pleasure in daily activities, change in weight or appetite, insomnia/hypersomnia, psychomotor retardation or agitation, loss of energy or fatigue, worthlessness or guilt, impaired concentration or indecisiveness, and thoughts of death or suicidal ideation/attempt (5). MDD is also associated with a significant reduction in life span, in particular due to the remarkable increase in vulnerability to chronic health conditions like cardiovascular disease (CVD) (6), autoimmune disease (7), and renal disease, as well as diabetes (8), and cancer (9). Moreover, MDD is highly comorbid with chronic medical conditions like inflammatory diseases (10), suggesting that inflammation might contribute to depression development and persistence.

Although the pathogenesis of MDD is yet unclear, depression and inflammation seem strictly intertwined and fueling each other (11). The co-occurrence of these two conditions has been hypothesized on the basis of different comorbidities, through a bidirectional link (12). In other words, depression seems to facilitate inflammatory responses, and inflammation promotes depression, with notable health consequences. Indeed, inflammation characterizes a number of disorders and systemic diseases, including CVD, diabetes, metabolic syndrome, rheumatoid arthritis, asthma, multiple sclerosis, chronic pain, and psoriasis, which, in turn, promote an elevated risk for depression (13, 14). In addition, individuals with chronic stress or mood disorders develop negative health behaviors (e.g., a sedentary lifestyle, poor diet, obesity, poor sleep, and smoking) that can lead to uncontrolled inflammation and depression (15).

Previous studies showed that – beyond specific illness risk – the link between depression and circulating inflammation has been associated with the risk of deaths (16) and hospitalizations (17) even predicting multiple re-hospitalizations (18). Different studies tested the role of inflammation in the relationship between depression and increased risk of mortality/hospitalizations. Hughes et al. (19) reported an association between depressive symptoms and all-cause mortality in an Irish prospective cohort [2,389 men, 54.7 (2.8) years], which was partly explained by increased C-reactive protein (CRP) levels. Likewise, in independent cohorts, inflammation partly mediated the influence of depressive symptoms on cardiovascular mortality (20) and hospitalization risk (21). Kop et al. found that elevated levels of inflammatory cytokines explained a small proportion (only 6.5%) of the incident CVD mortality associated with depression in a healthy cohort of U.S. elders (*N* = 907; 71.3 years). In line with this, in a longitudinal Australian cohort (*N* = 1,692; 55–85 years), the inflammatory markers interleukin-6 (IL-6) and CRP explained 10.9 and 8.1% of the risk of incident CVD hospitalizations associated with depression, respectively. Thus, although inflammation might contribute to the influence of depression on incident CVD, this mechanism accounts for a fairly small part of the total effect (21), supporting the idea that other pathways could be also involved in the depression–mortality link (22). Indeed, in a large longitudinal English cohort of older adults (*N* = 5,328; 52–89 years), clear sex-specific differences have been observed in the interrelationships between depressive symptoms, inflammation, and mortality (22). The authors observed that older men with both depressive symptoms and high levels of inflammation have an increased risk of CVD and all-cause mortality compared with men with depressive symptoms or inflammation alone; in addition, they reported independent influences of depressive symptoms and inflammation on mortality, finding no evidence of either moderation or mediation (22). In women, neither depressive symptoms nor inflammation predicted CVD or all-cause mortality (22).

More recently, in a longitudinal analysis of a large Italian population cohort, our group reported evidence suggesting not only potential mediation but also additive and interactive effects of circulating inflammation in the relationship between depression and increased hospitalizations/mortality risk, for all and specific (CVD) causes (17). In particular, we tested the role of INFLA score, a composite index based on four circulating markers [C-reactive protein (CRP), platelet count (Plt), white blood cell count (WBC), and granulocyte-to-lymphocyte ratio (GLR)] which tap into different components of the inflammatory process (23), allowing to evaluate combined effects of inflammation biomarkers. Still, we did not investigate effect modifications of gender, which would help clarifying the biological and social background of the relationship between depression, inflammation, and clinical risk. Similarly, the weight of somatic/neurovegetative (e.g., fatigue, psychomotor retardation, and altered appetite) and cognitive/psychological symptoms of depression (e.g., low mood, anhedonia, self-estimate, and suicidality) in this link is largely neglected, as well as the role of inflammation in these specific domains. Clarifying these aspects is of utter importance in light of two main findings: first, the association of somatic symptoms with circulating inflammation markers, consistently supported by independent studies (24–30), and second, a potential explanatory role of circulating inflammation in the relationship between the inflammatory potential of diet and the severity of depressive symptoms, which was prominent for somatic but not for cognitive symptoms, as supported by recent evidence (31). Here, we further explored the influence of depression severity on mortality and hospitalization risk for all and CVD causes by (i) analyzing potential effect modifications of gender, (ii) testing the independent influence of somatic and cognitive depressive symptoms on these clinical outcomes, and iii) examining in detail the potential mediation role of the single component biomarkers of INFLA score. The findings reported here allow to further disentangle the relationship between depression, chronic health conditions, and clinical risk in an Italian population cohort.

## Materials and methods

### Population of study

Analyses were performed using data from the Moli-sani study, a population-based cohort of 24,325 participants aged ≥ 35 years, randomly recruited among residents in the Molise region (central Italy) between 2005 and 2010. Participants with ongoing pregnancy, disturbances in understanding/willing processes, poly-traumas, or coma were excluded (32). The Moli-sani study was approved by the ethical committee of the Catholic University in Rome, Italy, and all the participants provided written informed consent.

For the present work, we selected participants for whom complete questionnaire data on depressive symptoms were available (*N* = 13,776). We removed participants with unreliable medical/dietary questionnaire data or blood marker levels (sum of leukocyte fractions < 99% or > 101%) or with signs of acute ongoing inflammation (high-sensitivity serum C-reactive protein levels ≥ 20 mg/L, as in (22). After these filters, 13,191 samples were available for subsequent analyses.

### Exposure measures: Depression forms and depressive symptom factors

Depressive symptoms were assessed through an alternative validated version of the PHQ-9 (17, 33). This scale (hereafter called PHQ9-6) adapted over eight items, due to the unavailability of data on the feeling of failure, but showed comparable accuracy and robustness in the classification of different depression forms (34). Participants were classified into three different classes based on severity of depressive symptoms, namely, no/minimal depression (PHQ9-6 ≤ 4), mild/moderate depression (4 < PHQ9-6 < 15), and severe depression (PHQ9-6 ≥ 15) (34).

To investigate specific domains of depressive symptoms, we derived latent variables tagging somatic (MR1) and cognitive symptoms (MR2), through a polychoric factor analysis, as described in (31). Briefly, using the psych package in R (see URLs), we first computed a polychoric correlation matrix of the available depression items and then derived two polychoric factors applying Oblimin (oblique) rotation. The derived factors – with a Pearson’s r correlation of 0.72 – showed prominent loadings of neurovegetative/somatic and cognitive/affective symptoms, explaining 35.9 and 17.1% of their shared variance (31). Specifically, MR1 showed moderate to high loadings of impaired movements/speaking and concentration (0.8), altered appetite/eating, tiredness/low energy (0.7), and altered sleeping (0.6), while MR2 showed a high loading of anhedonia (1) and moderate loadings of low mood and suicidal ideation (0.4). These factors were tested like the main exposure (i.e., depression severity) in all downstream analyses (see the following text).

### Outcome measures: Hospitalizations and death events

First hospital admissions data were obtained from the Molise regional registry of hospital discharge records, updated to 31 December 2015 (35).

A hospitalization was defined as any stay lasting ≥ 24 h in any hospital, clinic, emergency room, or similar. Primary and secondary diagnoses for hospitalization were coded using major diagnostic categories (MDCs) of the Italian Diagnosis-Related Groups classification (version 24) and the International Classification of Diseases, 9th version (ICD-9; see URLs), and the primary diagnosis was elected as a cause of hospitalization accordingly (35). Here, we focused on hospitalization events for all causes, excluding pregnancy complications, childbirth, rehabilitation, chemotherapy, and/or radiotherapy. Also, we analyzed hospitalizations for specific causes, such as cardiovascular disease (CVD, defined as primary diagnosis MDC: 05, or ICD-9: 390–459 and MDC: 01/05), ischemic heart disease (IHD, primary diagnosis ICD-9: 410–414, or surgical procedure with ICD-9: 360–361), and cerebrovascular events (primary diagnosis ICD-9: 430–434, 436–438, or surgical procedure with ICD-9: 381.2).

Overall and cause-specific mortality data till 31 December 2015 were obtained from the Italian mortality (ReNCaM) registry and with the Moli-sani database through unique identifier codes for each participant. Death events were validated by Italian death certificates (ISTAT form) and coded according to the ICD-9. Cardiovascular mortality was defined when the underlying cause of death included ICD-9 codes 390–459, respectively (36). Within cardiovascular mortality, ICD-9 codes 430–438 were used to define deaths due to cerebrovascular disease (i.e., both hemorrhagic and ischemic stroke), and codes 410–414 and 429 for deaths caused by ischemic heart disease (IHD) (36); then these were collapsed into a single mortality cause due to the paucity of events within each class (37). Non-cardiovascular causes of death were included in death events for all causes.

Following the aforementioned criteria, 13,176 participants were finally involved in the analysis of hospitalizations (4,856 all-cause events; median follow-up 7.28 years) and 13,164 subjects in the analysis of deaths (471 all-cause events; median follow-up 8.24 years).

### Statistical analyses

Statistical analyses were performed by SAS/STAT software, version 9.4 of the SAS System for Windows© 2009. SAS Institute Inc., and SAS is a registered trademark of SAS Institute Inc., Cary, NC, USA. All survival analyses were carried out through case-complete multivariable Cox proportional hazards (PH) regressions, incrementally adjusted for (i) sociodemographic covariates (age, educational attainment, and sex, where applicable), (ii) INFLA score, and (iii) lifestyle factors or their proxies (smoking habits, physical activity, body mass index, adherence to Mediterranean diet, and daily energy intake) and for the main chronic disease conditions (diabetes, history of CVD, and cancer) (17). A detailed description of these variables can be found in the Supplementary Methods.

First, we tested gender-by-depression severity interactive associations (in the total sample) and gender-stratified associations of depression severity with incident hospitalization risk and mortality, for all and specific (CVD) causes. Only significant associations detected both in interaction and in gender-stratified analyses were considered robust evidence of effect modification by gender. Then, we tested specific associations of the two polychoric factors tagging somatic and cognitive symptoms with the same outcomes in the total sample, reciprocally adjusting for the factor other than the exposure, in addition to the aforementioned mentioned covariates.

### Mediation analyses on inflammation markers

In our previous study, we tested INFLA score as a potential mediator of the association between depression severity and incident all-cause hospitalizations/mortality risk (17). INFLA score represents a global index capturing both serum and cellular circulating inflammation, based on high-sensitivity C-reactive protein serum (CRP) levels, blood platelet count (Plt), white blood cell count (WBC), and granulocyte-to-lymphocyte ratio (GLR) (23). Indeed, CRP is the most commonly used marker to evaluate systemic inflammation in humans because it is relatively stable and easy to measure (38). Increased Plt and WBC have been frequently studied as cellular inflammation indicators at the epidemiological level (39), as well as the neutrophil-to-lymphocyte ratio (NLR) (40). Here, we used the GLR as a proxy measure of the NLR since neutrophils represent the majority of granulocytes (∼95%), and the NLR showed a much higher missing rate than the GLR in our cohort (41).

To investigate circulating inflammation biomarkers that cause the significant mediation effects observed for INFLA score, we applied the %MEDIATE macro (42) to Cox PH models, predicting the incident risk of hospitalizations and mortality of all and CVD causes, testing each single component of INFLA score as a potential mediator. This analysis was carried out in the total sample, with models adjusted for age, sex, and education level as covariates so as to compare with our previous study (17). These analyses were also repeated using Cox PH regressions testing somatic and cognitive symptom factors as exposures, as well as using gender-stratified analyses.

## Results

A comparison of the population under study (13,191 participants passing) with the removed participants (*N* = 11,134) revealed a higher frequency of men (49 vs. 47%) and younger age (mean (SD) 53.2 (10.9) vs. 58.9 (12.4) years, *p* < 0.0001), as well as a generally higher education level (*p* < 0.0001, Table 1). The analyzed subset showed a lower prevalence of chronic health conditions like CVD, cancer, diabetes, hypertension, and hyperlipidemia (*p* < 0.0001). This was largely determined by the availability of psychometric assessment data in less than 14,000 participants and is probably due to the fact that people accepting to take self-administered psychological questionnaires are younger and more educated since these topics are not always easily understandable and are possibly considered less important by elders and less educated subjects (41).

**TABLE 1:** Baseline characteristics by gender of the analyzed Moli-sani population (*N* = 13,191).

Variable	Population under study (*N* = 13,191)	Removed participants (*N* = 11,134)	P for difference (analyzed vs. removed)	Women (*N* = 6,693)	Men (*N* = 6,498)	P for difference (women vs. men)
Sex (men)	6,498 (49.26%)	5,204 (46.74%)	<0.0001	–	–	–
Age (y)	53.15 (10.87)	58.91 (12.43)	<0.0001	52.46 (10.71)	53.84 (10.98)	<0.0001
***Education*** Primary or less Lower secondary Upper secondary Post-secondary	1,984 (15.06%) 3,919 (29.74%) 5,302 (40.23%) 1,973 (14.97%)	4,284 (38.57%) 2,823 (25.41%) 2,957 (26.62%) 1,044 (9.40%)	<0.0001	1,144 (17.10%) 1,895 (28.33%) 2,625 (39.24%) 1,025 (15.32%)	840 (12.94%) 2,024 (31.19%) 2,677 (41.25%) 948 (14.61%)	<0.0001
***Health conditions*** CVD Cancer Diabetes Hypertension Hyperlipidemia	594 (4.54%) 387 (2.94%) 498 (3.82%) 3,148 (24.01%) 863 (6.59%)	833 (7.61%) 401 (3.63%) 716 (6.53%) 3,839 (34.77%) 1048 (9.54%)	<0.0001 0.0026 <0.0001 <0.0001 <0.0001	161 (2.43%) 232 (3.48%) 169 (2.56%) 1,500 (22.54%) 366 (5.51%)	433 (6.72%) 155 (2.39%) 329 (5.10%) 1,648 (25.54%) 497 (7.70%)	<0.0001 0.0002 <0.0001 <0.0001 <0.0001
***Lifestyle factors*** Smokers Energy intake (Kcal/day) MeDi Score Physical activity (MET-h/day) BMI (kg/m^2^)	3,192 (24.22%) 2,139.7 (630.5) 4.36 (1.66) 3.38 (3.77) 27.72 (4.63)	2,390 (21.50%) 2,006.5 (702.7) 4.33 (1.62) 3.60 (4.30) 28.45 (4.92)	<0.0001 <0.0001 0.21 <0.0001 <0.0001	1,513 (22.63%) 1,954.9 (538.6) 4.23 (1.64) 2.52 (2.83) 27.30 (5.16)	1,679 (25.85%) 2,330 (661) 4.48 (1.66) 4.28 (4.36) 28.16 (3.96)	<0.0001 <0.0001 <0.0001 <0.0001 <0.0001
***Systemic inflammation*** INFLA-score CRP (mg/L) Plt (× 10^9^/L) WBC (× 10^9^/L) GLR	−0.20 (5.90) 2.27 (2.58) 251.9 (62.99) 6.22 (1.76) 1.98 (0.76)	0.05 (6.19) 2.98 (3.89) 245.20 (65.37) 6.25 (1.81) 2.06 (1.11)	0.001 <0.0001 <0.0001 <0.0001 <0.0001	0.05 (6.05) 2.38 (2.71) 266.2 (64.72) 5.96 (1.53) 2.00 (0.76)	−0.46 (5.73) 2.15 (2.43) 237.10 (57.56) 6.49 (1.92) 1.97 (0.74)	<0.0001 <0.0001 <0.0001 <0.0001 0.02
***Depression severity*** No/minimal Mild/moderate Severe	8,697 (65.93%) 4,272 (32.39%) 222 (1.68%)	333 (56.92%)^a^ 237 (40.51%)^a^ 15 (2.56%)^a^	<0.0001	3,764 (56.24%) 2,750 (41.09%) 179 (2.67%)	4,933 (75.92%) 1,522 (23.42%) 43 (0.66%)	<0.0001

Here, we report frequency (%) for categorical variables, or, alternatively, mean values and standard deviations (SD) for continuous variables.

*P*-values resulting from statistical comparisons of the analyzed vs. non-analyzed participants and of women vs. men are reported.

The chi-squared test was applied to education levels, CVD, cancer, smoking classes, Fisher exact test to sex, diabetes, and hypertension, unpaired t-test to age, INFLA score, Plt, GLR, MeDi score, caloric intake and BMI, and Wilcoxon signed rank test to CRP, WBC, and physical activity levels (see Supplementary Methods for further details on the definition of these covariates).

^a^ These figures refer only to removed participants with psychometric assessment available. BMI, body mass index; CVD, cardiovascular disease; MeDi, adherence score to Mediterranean diet (MeDi) (43); CRP, C-reactive protein; Plt, platelet count; WBC, white blood cell count; GLR, granulocyte-to-lymphocyte ratio.

Within the analyzed subset, women (*N* = 6,693) were younger [52.5 (10.7) vs. 53.8 (11.0) years] and less educated (*p* < 0.0001) and showed a lower prevalence of chronic health conditions like CVD, hypertension, and hyperlipidemia, and a higher prevalence of depression than men (*p* < 0.0001; Table 1).

### Effect modification of gender on the association between depression severity, hospitalization risk, and mortality

We later report associations from the baseline models (model 1), which showed stable effects across all incremental models tested. Gender-by-depression severity interaction was significantly associated with all-cause mortality (p for interaction 0.03), but not with hospitalization risks (Table 2). This evidence was robust across incremental models tested and supported by gender-stratified analyses, where we observed a significant association of moderate depression with death risk in women (HR [CI_95%_] = 1.60 [1.15; 2.22]), but not in men (1.02 [0.80; 1.31]; Supplementary Tables 1A, B). Conversely, in men, severe depression was associated with mortality more remarkably than in women, although formal tests of interaction were not statistically significant (Table 2).

**TABLE 2:** Associations of gender-by-depression severity interaction with incident hospitalization risk and mortality.

Event	Cause	N (events)	Depression severity (interaction with Gender)	P for interaction Model 1	P for interaction Model 2	P for interaction Model 3
Hospitalizations	All-cause	13,176 (4,856)	Moderate	0.47	0.47	0.61
			Severe	0.27	0.28	0.26
	CVD	13,176 (1,650)	Moderate	0.58	0.57	0.33
			Severe	0.61	0.61	0.66
	IHD	13,176 (459)	Moderate	0.45	0.43	0.19
			Severe	0.25	0.23	0.49
	CeVD	13,176 (226)	Moderate	0.45	0.47	0.61
			Severe	0.05	0.06	0.10
Deaths	**All-cause**	**13,164** (471)	**Moderate**	**0.03**	**0.02**	**0.01**
			Severe	0.34	0.40	0.58
	CVD	13,155 (141)	Moderate	0.19	0.16	0.18
			Severe	0.38	0.47	0.31
	IHD/CeVD	13,155 (79)	Moderate	0.13	0.11	0.49
			Severe	0.81	0.94	0.52

Associations of gender-by-depression severity interaction terms with risk of hospitalizations and deaths for all and specific causes are reported. Significant interactive associations (*p* < 0.05) are highlighted in bold. Final sample size (N) indicates the number of samples actually analyzed with a case-complete approach in model 1, after removing samples with missing covariates and/or event and follow-up data.

Legend: Model 1, age + education; model 2, model 1 + INFLA score; model 3, model 2 + lifestyles + clinical health conditions.

CVD, cardiovascular disease; IHD, ischemic heart disease; CeVD, cerebrovascular disease.

Similarly, a gender-stratified analysis revealed partly differential associations of depression severity with hospitalization risk (Supplementary Tables 2A, B): while in women, moderate and severe depression were associated with an increase in CVD hospitalization risk (HR = 1.36 [1.16; 1.60] and 2.03 [1.39; 3.00], respectively), and in men, only moderate depression was significantly associated with an increased risk (1.32 [1.15; 1.50]). However, severely depressed men showed a notably increased risk of hospitalization for cerebrovascular disease (6.82 [2.97; 15.70]), which was not observed in women (p for interaction 0.05; Table 2).

Of note, INFLA score was associated with a significant additive increase of hospitalization risk both in women (1%) and men (2%), where the increment was more pronounced for CVD (2%), IHD (3%), and CeVD hospitalizations (6%). While this increment was substantial for mortality in men (ranging between 3% for all-cause and 9% for IHD/cerebrovascular deaths), this was not significant in women (Supplementary Tables 3A, B). No significant interactive effects of depression severity and INFLA score on incident risk of hospitalizations and mortality were observed in both genders (Supplementary Tables 4A, B).

### Association of depressive symptom factors with hospitalization risk and mortality

The analysis of polychoric factors tagging somatic (MR1) and cognitive depressive symptoms (MR2) in multivariable models reciprocally adjusting for both factors revealed a significant association of the somatic factor with hospitalizations for all (1.16 [1.11; 1.20]) and CVD causes (1.14 [1.07; 1.21]), while associations with IHD and CeVD events were significant but not stable across all incremental models tested (Table 3A). Conversely, we detected no significant association for any of the factors tested with mortality (Table 3B). When modeled jointly with polychoric factors, INFLA score showed small but significant independent increases in the risk of hospitalizations (1% for all, 2% for CVD, 3% for IHD, and 4% for CeVD causes) and of deaths (3% for all, 6% for CVD, and for IHD/CeVD causes) (Supplementary Tables 5A, B). Again, INFLA score did not reveal any significant interactive influence with both polychoric factors on the clinical outcomes analyzed (Supplementary Table 6).

**TABLE 3:** Associations of depressive symptom factors with incident **(A)** hospitalization risk and **(B)** mortality.

(A)					

Cause of hospitalization	N (events)	Polychoric factor	Model 1	Model 2	Model 3
					
			HR [95% CI] (*P*-value)	HR [95% CI] (*P*-value)	HR [95% CI] (*P*-value)
All causes	13,168 (4,855)	MR1 + MR2	**1.16 [1.11; 1.20]** **(< 0.0001)**	**1.15 [1.11; 1.20]** **(< 0.0001)**	**1.13 [1.09; 1.18]** **(< 0.0001)**
			0.98 [0.94; 1.02] (0.23)	0.98 [0.94; 1.02] (0.25)	0.98 [0.94; 1.02] (0.26)
CVD	13,168 (1,649)	MR1 + MR2	**1.14 [1.07; 1.21]** **(0.0001)**	**1.13 [1.06; 1.21]** **(0.0002)**	**1.09 [1.02; 1.17]** **(0.01)**
			1.023 [0.96; 1.09] (0.50)	1.02 [0.96; 1.09] (0.48)	1.02 [0.96; 1.10] (0.50)
IHD	13,168 (459)	MR1 + MR2	**1.16 [1.02; 1.32]** **(0.02)**	**1.15 [1.01; 1.31]** **(0.03)**	1.05 [0.92; 1.20] (0.47)
			0.97 [0.85; 1.10] (0.63)	0.97 [0.85; 1.10] (0.61)	0.96 [0.84; 1.09] (0.51)
CeVD	13,168 (226)	MR1 + MR2	1.08 [0.91; 1.28] (0.40)	1.07 [0.90; 1.27] (0.43)	1.08 [0.91; 1.29] (0.37)
			**1.20 [1.02; 1.41]** **(0.03)**	**1.20 [1.02; 1.41]** **(0.03)**	1.18 [1.00; 1.40] (0.05)

**(B)**					

**Cause of death**	**N (events)**	**Polychoric factor**	**Model 1**	**Model 2**	**Model 3**
					
			**HR [95% CI]** **(*P*-value)**	**HR [95% CI]** **(*P*-value)**	**HR [95% CI]** **(*P*-value)**

All causes	13,156 (471)	MR1 + MR2	1.05 [0.93; 1.18] (0.47)	1.04 [0.92; 1.18] (0.49)	1.04 [0.91; 1.18] (0.54)
			1.11 [0.99; 1.25] (0.08)	1.10 [0.98; 1.24] (0.10)	1.06 [0.94; 1.20] (0.36)
CVD	13,147 (141)	MR1 + MR2	1.12 [0.90; 1.39] (0.32)	1.12 [0.90; 1.39] (0.32)	1.14 [0.90; 1.45] (0.27)
			1.05 [0.85; 1.29] (0.67)	1.03 [0.84; 1.28] (0.77)	0.97 [0.77; 1.23] (0.80)
IHD/CeVD	13,147 (79)	MR1 + MR2	1.18 [0.88; 1.58] (0.28)	1.17 [0.87; 1.58] (0.30)	1.18 [0.85; 1.63] (0.32)
			0.94 [0.70; 1.26] (0.68)	0.93 [0.69; 1.25] (0.62)	0.85 [0.61; 1.19] (0.34)

Hazard ratio (HR), relevant confidence interval (95% CI), and corresponding *p*-values for polychoric factors tagging somatic (MR1) and cognitive symptoms (MR2) are reported, modeled both separately and jointly in a multivariable Cox PH regression. Significant HRs (*p* < 0.05) are highlighted in bold. Final sample size (N) indicates the number of samples actually analyzed with a case-complete approach in model 1, after removing samples with missing covariates and/or event and follow-up data. Legend: model 1, age + sex + education; model 2, model 1 + INFLA score; model 3, model 2 + lifestyles + clinical conditions. CVD, cardiovascular disease; IHD, ischemic heart disease; CeVD, cerebrovascular disease. Sample size and number of events may vary compared with Table 2, depending on the availability of the other variables involved in the analysis.

### Role of single inflammation markers in the association between depression and clinical outcomes

We report here point estimates of the exposure effect on the outcome (PTE) explained by the intermediate variable tested, with relevant *p*-values, as per %MEDIATE macro applied to minimal models including age, sex, and education as covariates [model 1, as in (17)], unless otherwise stated.

In the association between depression severity and all-cause hospitalizations, a dissection of the significant PTE of INFLA score revealed prominent explanatory effects of the leukocyte components, with WBC and GLR explaining 1.5% (*p* = 0.02) and 2.0% (*p* = 3 × 10^–3^) of the association, respectively. These effects were generally confirmed when we analyzed hospitalizations for CVD causes, and even increased for WBC (Table 4A). The latter explained 2.4% of the influence on all CVD hospitalizations (*p* = 0.01), increasing up to 5.3% (*p* = 0.01) and 5.7% (*p* = 7 × 10^–3^) for IHD and cerebrovascular events, respectively. Among non-leukocytic components of circulating inflammation, CRP explained 4.6% of the associations between depression severity and IHD events (*p* = 0.048). As for mortality risk, we detected a significant explanatory role for both WBC (4.8%, *p* = 0.03) and GLR (11.2%, *p* = 0.02). Again, PTEs further increased when we analyzed CVD (13.7 and 10.4%) and IHD/CeVD events (23.9 and 16.3%) but remained significant only for WBC (Table 4B).

**TABLE 4:** Proportion of the association of depression severity with incident **(A)** hospitalization risk and **(B)** mortality explained by INFLA score component biomarkers.

(A)						

Cause of hospitalization	N (events)	INFLA-score (17)	CRP	Plt	WBC	GLR
All causes	12,746 (4,640)	**2.1% (7 × 10^–3^)**	<1% (NS)	<1% (NS)	**1.5% (0.02)**	**2.0% (3 × 10^–3^)**
CVD	12,746 (1,560)	**3.0% (3 × 10^–3^)**	1.0% (0.12)	<1% (NS)	**2.4% (0.01)**	**2.0% (0.03)**
IHD	12,746 (431)	**6.8% (6 × 10^–3^)**	**4.6% (0.048)**	<1% (NS)	**5.3% (0.01)**	<1% (NS)
CeVD	12,746 (214)	**5.1% (0.01)**	<1% (NS)	<1% (NS)	**5.7% (7 × 10^–3^)**	4.1% (0.05)

**(B)**						

**Cause of death**	**N (events)**	**INFLA-score (17)**	**CRP**	**Plt**	**WBC**	**GLR**

All	12,735 (440)	**8.0% (7 × 10^–3^)**	2.9% (0.18)	<1% (NS)	**4.8% (0.03)**	**11.2% (0.02)**
CVD	12,733 (125)	**13.7% (0.02)**	1.2% (0.37)	<1% (NS)	**13.7% (0.01)**	10.4% (0.11)
IHD/CeVD	12,733 (69)	**29.9% (0.03)**	3.3% (0.34)	1.9% (0.32)	**23.9% (0.02)**	16.3% (0.11)

Percentage of total effect (PTE) and relevant *p*-values as produced by the %MEDIATE macro are reported for each inflammation biomarker, in multivariable Cox PH models modeling incident (a) hospitalizations and (b) mortality risk as a function of depression severity, adjusted for sociodemographic covariates (age, sex, and education). Significant PTEs (*p* < 0.05) are highlighted in bold. CRP, C-reactive protein (mg/L, log scale); Plt, platelet count (× 10^9^/L); WBC, white blood cell count (× 10^9^/L, log scale); GLR, granulocyte-to-lymphocyte ratio; CVD, cardiovascular disease; IHD, ischemic heart disease; CeVD, Cerebrovascular disease; NS, non-significant (PTE too low to estimate a reliable *p*-value). Sample size and number of events may vary compared with Table 2, depending on the availability of the other variables involved in the analysis.

Trends similar to those observed in the total sample were observed in the gender-stratified analysis, but only in men, where INFLA score explained 3% of the influence of depression severity on all-cause mortality (*p* = 0.02), which increased to 5.5% (*p* = 0.007) for CVD, 11% (*p* = 0.01) for IHD, and 8.7% (*p* = 0.006) for CeVD deaths. PTEs were even higher for hospitalizations, namely, 20.5% (*p* = 0.01) for all-cause mortality and 24.5% (*p* = 0.01) for CVD events. No significant PTEs were observed for INFLA score in women (Supplementary Tables 7A, B). PTEs of INFLA score in the association of depressive symptom factors with clinical outcomes – adjusting for the other factor not used as exposure – were significant only for the somatic symptom factor (MR1) and for hospitalization risk. INFLA score explained 2.5% (*p* = 0.006) of the association with all-cause hospitalizations, and 4.1 and 8.6% of the associations with CVD and IHD hospitalizations risk (*p* < 0.05). Significant PTEs were observed neither for mortality nor for associations with MR2 (Supplementary Tables 8A, B).

## Discussion

In this study, we untangled the potential role of circulating inflammation in the link of depression with mortality and hospitalization risk for all and specific causes, which we demonstrated in a previous study (17). In that study, we reported significant proportions of the influence of depression on clinical risks – ∼30% of coronary heart disease/stroke mortality and ∼3–8% of CVD hospitalizations to be explainable by INFLA score, a composite marker of low-grade inflammation. In the current study, we observed that leukocyte-related components like total counts and granulocyte-to-lymphocyte ratio explained a small but significant proportion of effect on mortality and hospitalization risks, which was especially pronounced for CVD causes. These proportions ranged from 11% (for GLR on all-cause mortality) to 24% (for WBC on IHD/CeVD mortality) and are comparable to (and sometimes higher than) those observed for INFLA score in our previous work (17). Overall, this evidence suggests that cellular components of inflammation may represent a more direct mediation pathway between mental health and clinical risks, potentially explaining at least part of the bidirectional link between mental and cardiovascular health (6).

White blood cell count has been reported to fully mediate the relationship between depressive symptoms and all-cause mortality in a recent Chinese study (*N* = 4,053; ≥ 60 years) (44), although contrasting evidence links WBC with depression risk. In a patient cohort from the United States [*N* = 2,400; 54.1 (16.8) years], those with low WBC levels had a greater risk of subsequent hospitalization with depression (45), while in a large study including patients with bipolar disorder from two clinical trials in the United States (*N* = 765; 18–62 years), those with both high and low WBC levels showed increased symptom severity and specific clusters of symptoms, which differed depending on gender but were most pronounced among men (46).

The neutrophil-to-lymphocyte ratio (NLR), representing the innate immunity component of inflammation (47) and tagged here by GLR, has also been proposed as a useful biomarker to predict cardiovascular risk (47, 48), poor outcomes in cardiovascular diseases, and all-cause mortality (47, 49, 50). Recent studies showed that the NLR is a good indicator of inflammation state and is higher in patients with MDD (51, 52), a finding further corroborated by evidence that the NLR is positively correlated with severity of depression (53). Suicidal depressive patients also have higher NLRs than both non-suicidal patients and healthy controls, suggesting that inflammation may also play a central role in the pathogenesis of suicidal behavior in MDD (54).

Of interest, CRP explained a non-negligible proportion (4.6%) of the influence of depression severity only on IHD hospitalization risk, a finding not that surprising, in light of contrasting results on its involvement in the link between depressive symptoms and all-cause or CVD mortality and hospitalization risk. Indeed, some previous studies detected a small but significant mediating role of cytokine-related inflammatory biomarkers (i.e., CRP and IL-6) in the link between depressive symptoms and all-cause or CVD mortality (19, 20), which was slightly higher for CVD hospitalization outcomes (21, 55). However, Lawes et al. observed no significant mediation effect of inflammation between depression and all-cause/CVD mortality in both genders (22), while Davidson et al. observed that depressive symptoms were associated with an increased risk of incident coronary heart disease (CHD) events, in a way independent of both traditional risk factors and inflammatory biomarkers, particularly CRP levels (56). Overall, these data suggest that classical inflammatory biomarkers only partially explain the association between depression and clinical (in particular cardiovascular) risks, at best, and warrant further research on other inflammatory markers and alternative mediation pathways, which may help filling the gap.

Significant gender-specific differences were observed in the relationship between depression severity and mortality, but not hospitalization risk. Moderately depressed women showed increased all-cause death incidence, while in men, severe depression was associated with mortality of all and specific (IHD/CeVD) causes. This evidence seems concordant with the scarce literature on the topic (22, 57), and may be due to the trend in men not to report milder symptoms or seek treatment until depressive symptoms are more severe (58), which may result in lower power in the comparison between non- and mildly depressed subjects. Similarly, the role that INFLA score played in these associations was more pronounced for men, both as an additive term and as a potential mediator.

The present study also showed that mostly somatic depressive symptoms were associated with hospitalizations for all and CVD causes, in line with previous reports of these symptoms better predicting health status and cardiovascular prognosis in patients with cardiovascular disease (59, 60), although another study showed comparable associations with incident cardiac events between symptom domains (61). Moreover, we observed that a small but significant proportion of the association between the somatic symptom factor and hospitalization risks was explained by INFLA score, while associations with the cognitive factor were not, in line with recent evidence in another large British population cohort (62). This suggests that inflammation may play a minor role in mediating the effects of depression on cardiac events, especially when considering cognitive affective symptoms (63). Again, further research is warranted to clarify the differential associations between somatic and cognitive symptoms, especially in population settings, and substantiate the role of inflammation in these links.

### Strengths and limitations

This study presents some strengths. First, to our knowledge, no study so far has investigated cellular inflammatory components of inflammation as possible mediators in the link between depression and all-cause/CVD mortality and hospitalization. Second, the advantage of a large homogeneous population in the targeted analysis on each single component biomarker of INFLA score allowed detecting novel explanatory effects of the link between depression and clinical risks, which were never observed before in the field. Third, the analysis of two factors tagging different depressive domains, such as somatic and cognitive symptoms, and of interactions with gender allowed to further untangle this association, identifying potential gender-specific mechanisms. Although independent studies previously used factor analysis applied to depressive symptoms to analyze their relationship with inflammatory markers and arterial stiffness (e.g., 24, 25, 64), we are not aware of any study using this approach to analyze the link among depression, inflammation, later risk of hospitalization, and death. Therefore, further studies are warranted to substantiate this approach and the reported findings.

Limitations of our study should also be noted. The simultaneous assessment of depressive symptoms and inflammatory markers did not allow us to establish clear directionality in the mediation analysis. The lack of an item in the PHQ-9 assessment represents a further limitation in defining depression severity classes, although this subscale already showed comparable performance (34) and the polychoric factor analysis of depressive symptoms should have minimized the resulting bias. Moreover, owing to the observational design, we cannot fully rule out the potential of residual confounding by unmeasured factors. Data on depressive symptoms are self-reported, and this could lead to misclassification of exposures. In addition, the lack of precise information on medical treatments for some health conditions (e.g., CVD and cancer) makes it difficult to estimate the effect these may have on our findings. Finally, data were measured at baseline only; thus, potential changes occurred over life course might have modified the strength of the findings.

## Conclusion

**FIGURE 1:**
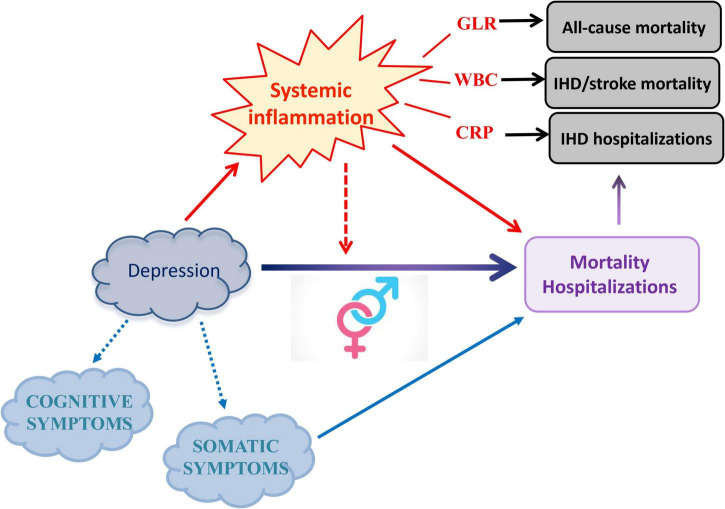
Main hypothesis investigated and evidence observed in the present study. Here, we untangled the role of specific circulating inflammatory markers in the influence of depression severity on mortality and hospitalization risk, further elucidating differential associations within gender strata and with depressive symptom domains. Figure adapted from 31.

In conclusion, this study suggests a prominent explanatory role of cellular components of inflammation, in particular granulocytes and lymphocytes, in the relationship between depression severity and mortality/hospitalization risk for all and CVD causes. Moreover, it sheds light on the depression–inflammation–cardiovascular disease pathway, highlighting the importance of gender and of somatic depressive symptoms in this link (Figure 1). However, much remains to be done to clarify the relationship between immunity, depression, and its comorbidities like cardiovascular disorders in detail, especially to enlighten their shared genetic and epigenetic underpinnings and the effect of medications for chronic health conditions on this complex interplay. Future studies should be aimed to clarify these aspects and identify gender- and symptom-specific mechanisms.

## Data availability statement

The datasets presented in this study can be found in online repositories. The names of the repository/repositories and accession number(s) can be found below: https://repository.neuromed.it/. Credentials are available upon request to the corresponding author (alessandro.gialluisi@gmail.com).

## Ethics statement

The studies involving human participants were reviewed and approved by the Catholic University of Rome. The patients/participants provided their written informed consent to participate in this study.

## Author contributions

AG and LI conceived the study. ADeC and SM carried out biological sample management and measurements. SC and ADi performed the statistical data elaboration and curation in the Moli-sani study. AG, FS, and SO analyzed the data. FB carried out psychometric assessment. AG and FB wrote the first draft of the manuscript, with contributions and critical review from all the co-authors. LI, MD, ADi, CC, and GG originally inspired the Moli-sani study.
