# Supplementary material for: Role of leukocytes, gender, and symptom domains in the influence of depression on hospitalization and mortality risk: Findings from the Moli-sani study
Source: Front Psychiatry. 2022 Oct 13;13:959171. doi: 10.3389/fpsyt.2022.959171 (PMC9606761; doi:10.3389/fpsyt.2022.959171)
Supplement: Supplementary file 1 [file Data_Sheet_1.docx]

**Supplementary Methods**

***Blood measurements***

Blood samples were obtained between 7 and 9 AM from participants who had fasted overnight and had refrained from smoking for at least 6 h. Biochemical analyses were performed in the centralised Moli-sani laboratory (Santimone et al., 2011). All haemochromocytometric analyses (white blood cell and platelet counts, granulocyte % and lymphocyte %) were performed by cell counter (Coulter HMX, Beckman Coulter, Milan, Italy) within 3 hours (h) from venipuncture. High sensitivity C-reactive protein (CRP) was measured in fresh serum, by a latex particle-enhanced immunoturbidimetric assay (ILab 350 Instrumentation Laboratory, Milan, Italy).

***Definition of covariates***

*Education level* of participants was classified into four categories: *Primary, Lower secondary, Upper secondary* and *Post-secondary*.

Prevalent *diabetes* was defined as a dichotomous variable (Yes/No), based on the reported and verified use of specific drugs for the treatment of this disorder.

Prevalent *cardiovascular disease (CVD)* and *cancer* were defined as binary variables, classified into subjects reporting medical history of the disease (possibly supported by medical documentation or by the use of specific drugs), and those with no medical history of the disease.

*Smoking status* of participants was classified into three categories: *smokers, ex-smokers* (i.e. subjects who quitted at least one year before the interview) and *non-smokers*.

Leisure-time *physical activity* was assessed through a structured questionnaire and expressed as daily energy expenditure in metabolic equivalent task-hours (MET-h/day) (Ainsworth et al., 2000).

Food intake was assessed through the validated Italian EPIC food frequency questionnaire (Pisani et al., 1997). Adherence to *Mediterranean Diet* was defined according to the Mediterranean Diet (MeDi) Score (Trichopoulou et al., 2003). The EPIC questionnaire also allowed to compute the daily *energy intake* for the subjects assessed (Kcal/day), as well as alcohol consumption habits, along with a few additional questions (see (Costanzo et al., 2019) for details).

Height and weight were measured for each participant, hence *body mass index (BMI)* was calculated as kg/m^2^.

Over 13,191 subjects passing QC, we observed 6 participants with missing values for BMI, 13 for e*ducational attainment*, 11 for *smoking status*, 115 for *CVD*, 138 for *diabetes* and 34 for *cancer*. No missing data were observed for *sex*, *age*, *MeDi score*, *caloric intake* and *physical activity*. These participants were removed in the case-complete analysis, leaving 13,176 subjects for the analysis of hospitalizations and 13,164 subjects for the analysis of deaths (since 12 additional participants had missing time-to-death information).

***Computation of INFLA-score***

INFLA-score is global index conceived to capture both serum and cellular circulating inflammation based on four circulating biomarkers, namely C-reactive protein levels (CRP), blood platelet count (Plt), white blood cell count (WBC) and granulocyte-to-lymphocyte ratio (GLR) (Pounis et al., 2016). CRP is the most commonly used marker to evaluate systemic inflammation in humans, because it is relatively stable and easy to measure (Danesh et al., 2004); increased Plt and WBC have been frequently studied as cellular inflammation indicators at the epidemiological level (Bonaccio et al., 2014), as well as neutrophil–to-lymphocyte ratio (NLR) (Guthrie et al., 2013). Here, we used GLR as a proxy measure of NLR since neutrophils represent the majority of granulocytes (~95%) and NLR showed a much higher missing rate than GLR in our cohort (8,145 vs 2,143, over a total of 24,325 participants)(Gialluisi et al., 2020).

The INFLA-score consists of an equal weight combination of the four component biomarkers, as described in (Pounis et al., 2016). Briefly, for each biomarker, the distribution was divided into 10-tiles and each 10-tile was assigned a corresponding score, as follows: samples with a value laying in Q5 and Q6 (median values) were labelled with score 0, values >Q6 (higher tail of distribution) were assigned an increasing score from +1 (Q7) through +4 (Q10), while values <Q5 (lower tail of distribution) were assigned a negative score from -4 (Q1) to -1 (Q4). Then the INFLA-score was computed as a sum of the resulting scores over the four biomarkers tested, with total values ranging between -16 (lowest grade of inflammation) and +16 (highest grade of inflammation).

**Supplementary Results**

**Table S1.** Gender-stratified association analysis of depression severity with incident mortality for **a)** women and **b)** men.

**a)**

| Cause of death | N (events) | Depression form | Model 1 | Model 2 | Model 3 |
| --- | --- | --- | --- | --- | --- |
|  |  |  | HR [95% CI]  (p-value) | HR [95% CI]  (p-value) | HR [95% CI]  (p-value) |
| All causes | 6,685  (156) | **Moderate** | **1.60 [1.15; 2.22]**  **(0.005)** | **1.59 [1.14; 2.20]**  **(0.006)** | **1.64 [1.16; 2.31]**  **(0.005)** |
|  |  | Severe | 1.94 [0.88; 4.24]  (0.10) | 1.92 [0.88; 4.20]  (0.10) | 1.85 [0.79; 4.34]  (0.16) |
| CVD | 6,683  (48) | Moderate | 1.60 [0.88; 2.89]  (0.12) | 1.59 [0.87; 2.88]  (0.13) | 1.58 [0.79; 3.13]  (0.19) |
|  |  | Severe | 2.14 [0.49; 9.28]  (0.31) | 2.14 [0.49; 9.30]  (0.31) | 1.39 [0.18; 10.96]  (0.76) |
| IHD/CeVD | 6,683  (26) | Moderate | 1.42 [0.63; 3.21]  (0.40) | 1.42 [0.63; 3.21]  (0.40) | 0.89 [0.35; 2.30]  (0.81) |
|  |  | Severe | 3.65 [0.79; 16.80]  (0.10) | 3.65 [0.79; 16.80]  (0.10) | 1.81 [0.21; 15.60]  (0.59) |

**b)**

| **Cause of death** | **N (events)** | **Depression form** | **Model 1** | **Model 2** | **Model 3** |
| --- | --- | --- | --- | --- | --- |
|  |  |  | **HR [95% CI]**  **(p-value)** | **HR [95% CI]**  **(p-value)** | **HR [95% CI]**  **(p-value)** |
| All causes | 6,479  (315) | Moderate | 1.02 [0.80; 1.31]  (0.85) | 1.00 [0.78; 1.27]  (0.98) | 0.96 [0.74; 1.23]  (0.72) |
|  |  | **Severe** | **3.21 [1.59; 6.52]**  **(0.001)** | **2.95 [1.45; 5.98]**  **(0.003)** | **2.28 [1.07; 4.88]**  **(0.03)** |
| CVD | 6,472  (93) | Moderate | 1.11 [0.71; 1.73]  (0.64) | 1.04 [0.67; 1.63]  (0.85) | 1.00 [0.63; 1.60]  (0.99) |
|  |  | **Severe** | **4.35 [1.36; 13.89]**  **(0.01)** | **3.63 [1.13; 11.67]**  **(0.03)** | **3.30 [1.01; 10.74]**  **(<0.05)** |
| IHD/CeVD | 6,472  (53) | Moderate | 0.76 [0.40; 1.44]  (0.40) | 0.70 [0.37; 1.33]  (0.27) | 0.76 [0.39; 1.46]  (0.41) |
|  |  | **Severe** | **4.47 [1.08; 18.60]**  **(0.04)** | 3.60 [0.86; 15.00]  (0.08) | 3.50 [0.81; 15.10]  (0.09) |

Hazard Ratios (HR), relevant Confidence Intervals (95% CI) and corresponding p-values for moderately- and severely-depressed subjects (compared to non-depressed) are reported. Significant HRs (p<0.05) are highlighted in bold. Final sample size (N) indicates the number of samples actually analysed with a case-complete approach in Model 1, after removing samples with missing covariates and/or event and follow-up data.

Legend: Model 1: age + education; Model 2: Model 1 + INFLA-score; Model 3: Model 2 + lifestyles + clinical conditions. Abbreviations: CVD = cardiovascular disease; IHD = ischemic heart disease; CeVD = Cerebrovascular disease.

**Table S2.** Gender-stratified association analysis of depression severity with incident hospitalization risk, for **a)** women and **b)** men.

**a)**

| **Cause of hospitalization** | **N (events)** | **Depression form** | **Model 1** | **Model 2** | **Model 3** |
| --- | --- | --- | --- | --- | --- |
|  |  |  | **HR [95% CI]**  **(p-value)** | **HR [95% CI]**  **(p-value)** | **HR [95% CI]**  **(p-value)** |
| All causes | 6,689  (2,239) | **Moderate** | **1.22 [1.13; 1.33]**  **(<.0001)** | **1.22 [1.12; 1.33]**  **(<.0001)** | **1.19 [1.09; 1.30]**  **(<.0001)** |
|  |  | **Severe** | **1.50 [1.19; 1.89]**  **(0.0005)** | **1.50 [1.19; 1.88]**  **(0.0005)** | **1.38 [1.09; 1.75]**  **(0.008)** |
| CVD | 6,689  (630) | **Moderate** | **1.36 [1.16; 1.60]**  **(0.0002)** | **1.36 [1.16; 1.60]**  **(0.0002)** | **1.29 [1.09; 1.52]**  **(0.003)** |
|  |  | **Severe** | **2.03 [1.39; 3.00]**  **(0.0002)** | **2.01 [1.38; 2.94]**  **(0.0003)** | **1.90 [1.27; 2.84]**  **(0.002)** |
| IHD | 6,689  (101) | Moderate | 1.47 [0.98; 2.20]  (0.07) | 1.45 [0.96; 2.17]  (0.07) | 1.29 [0.84; 1.98]  (0.25) |
|  |  | **Severe** | **2.80 [1.19; 6.61]**  **(0.02)** | **2.77 [1.17; 6.52]**  **(0.02)** | 2.02 [0.77; 5.29]  (0.15) |
| CeVD | 6,689  (88) | Moderate | 1.13 [0.73; 1.73]  (0.59) | 1.12 [0.73; 1.73]  (0.59) | 1.10 [0.71; 1.72]  (0.67) |
|  |  | Severe | 2.00 [0.79; 5.07]  (0.14) | 2.00 [0.79; 5.06]  (0.14) | 2.14 [0.83; 5.53]  (0.11) |

**b)**

| **Cause of hospitalization** | **N (events)** | **Depression form** | **Model 1** | **Model 2** | **Model 3** |
| --- | --- | --- | --- | --- | --- |
|  |  |  | **HR [95% CI]**  **(p-value)** | **HR [95% CI]**  **(p-value)** | **HR [95% CI]**  **(p-value)** |
| All causes | 6,487  (2,617) | **Moderate** | **1.25 [1.15; 1.37]**  **(<.0001)** | **1.24 [1.14; 1.36]**  **(<.0001)** | **1.22 [1.12; 1.33]**  **(<.0001)** |
|  |  | **Severe** | **1.87 [1.28; 2.74]**  **(0.001)** | **1.85 [1.27; 2.71]**  **(0.001)** | **1.77 [1.20; 2.62]**  **(0.004)** |
| CVD | 6,487  (1,020) | **Moderate** | **1.32 [1.15; 1.50]**  **(<.0001)** | **1.30 [1.13; 1.48]**  **(0.0002)** | **1.19 [1.03; 1.36]**  **(0.017)** |
|  |  | Severe | 1.71 [0.94; 3.10]  (0.08) | 1.68 [0.93; 3.05]  (0.09) | 1.55 [0.83; 2.90]  (0.17) |
| IHD | 6,487  (358) | Moderate | 1.23 [0.98; 1.54]  (0.07) | 1.21 [0.96; 1.51]  (0.11) | 1.00 [0.79; 1.26]  (0.98) |
|  |  | Severe | 1.14 [0.37; 3.57]  (0.82) | 1.09 [0.35; 3.40]  (0.89) | 1.03 [0.33; 3.24]  (0.96) |
| CeVD | 6,487  (138) | Moderate | 1.41 [0.98; 2.02]  (0.06) | 1.35 [0.94; 1.93]  (0.10) | 1.37 [0.95; 1.99]  (0.09) |
|  |  | **Severe** | **6.82 [2.97; 15.70]**  **(<.0001)** | **6.13 [2.66; 14.10]**  **(<.0001)** | **6.53 [2.80; 15.20]**  **(<.0001)** |

Hazard Ratios (HR), relevant Confidence Intervals (95% CI) and corresponding p-values for moderately- and severely-depressed subjects (compared to non-depressed) are reported. Significant HRs (p<0.05) are highlighted in bold. Final sample size (N) indicates the number of samples actually analysed with a case-complete approach in Model 1, after removing samples with missing covariates and/or event and follow-up data.

Legend: Model 1: age + education; Model 2: Model 1 + INFLA-score; Model 3: Model 2 + lifestyles + clinical health conditions. Abbreviations: CVD = cardiovascular disease; IHD = ischemic heart disease; CeVD = Cerebrovascular disease.

**Table S3.** Gender-stratified additive association of INFLA-score with incident risk of hospitalizations and mortality for **a)** women and **b)** men.

**a)**

| **Cause of event** | **Effect on hospitalizations**  **HR [95% CI]**  **(p-value)** | **Effect on mortality**  **HR [95% CI]**  **(p-value)** |
| --- | --- | --- |
| **All-cause** | **1.01[1.00; 1.02]**  **(0.003)** | 1.02[0.99; 1.05]  (0.16) |
| CVD | 1.01[1.00; 1.02]  (0.22) | 1.03[0.98; 1.08]  (0.24) |
| IHD | 1.03[0.99; 1.06]  (0.12) | 1.01[0.94; 1.08]  (0.84) |
| CeVD | 1.00[0.97; 1.04]  (0.92) |  |

**b)**

| **Cause of event** | **Effect on hospitalizations**  **HR [95% CI]**  **(p-value)** | **Effect on mortality**  **HR [95% CI]**  **(p-value)** |
| --- | --- | --- |
| **All-cause** | **1.02[1.01; 1.02]**  **(<.0001)** | **1.03[1.01; 1.05]**  **(0.0007)** |
| **CVD** | **1.02[1.01; 1.03]**  **(<.0001)** | **1.06[1.02; 1.10]**  **(0.001)** |
| **IHD** | **1.03[1.01; 1.05]**  **(0.001)** | **1.09[1.04; 1.14]**  **(0.0006)** |
| **CeVD** | **1.06[1.03; 1.09]**  **(<.0001)** |  |

We report Hazard Ratios (HR) of hospitalizations and deaths for all and specific causes, with 95% Confidence Interval (CI) and relevant p-values, associated with a unit increase in INFLA-score, in additive models with depression severity (Model 2). Significant HRs (p < 0.05) are highlighted in bold. Abbreviations: CVD = cardiovascular disease; IHD = ischemic heart disease; CeVD = Cerebrovascular disease.

**Table S4.** Gender-stratified interactive effects of depression severity and INFLA-score on incident risk of hospitalizations and mortality, for **a)** women and **b)** men.

**a)**

| **Event** | **Cause** | **N (events)** | **Depression severity**  **(interaction with INFLA-score)** | **p for interaction** |
| --- | --- | --- | --- | --- |
| Hospitalizations | All-cause | 6,689  (2,239) | Moderate | 0.18 |
|  |  |  | Severe | 0.26 |
|  | CVD | 6,689  (630) | Moderate | 0.89 |
|  |  |  | Severe | 0.96 |
|  | IHD | 6,689  (101) | Moderate | 0.63 |
|  |  |  | Severe | 0.81 |
|  | CeVD | 6,689  (88) | Moderate | 0.54 |
|  |  |  | Severe | 0.19 |
| Deaths | All-cause | 6,685  (156) | Moderate | 0.68 |
|  |  |  | Severe | 0.12 |
|  | CVD | 6,683  (48) | Moderate | 0.18 |
|  |  |  | Severe | 0.83 |
|  | IHD/CeVD | 6,683  (26) | Moderate | 0.96 |
|  |  |  | Severe | 0.88 |

**b)**

| **Event** | **Cause** | **N (events)** | **Depression severity**  **(interaction with INFLA-score)** | **p for interaction** |
| --- | --- | --- | --- | --- |
| Hospitalizations | All-cause | 6,487  (2,617) | Moderate | 0.35 |
|  |  |  | Severe | 0.93 |
|  | CVD | 6,487  (1,020) | Moderate | 0.71 |
|  |  |  | Severe | 0.23 |
|  | IHD | 6,487  (358) | Moderate | 0.41 |
|  |  |  | Severe | 0.56 |
|  | CeVD | 6,487  (138) | Moderate | 0.99 |
|  |  |  | Severe | 0.82 |
| Deaths | All-cause | 6,479  (315) | Moderate | 0.99 |
|  |  |  | Severe | 0.36 |
|  | CVD | 6,472  (93) | Moderate | 0.84 |
|  |  |  | Severe | 0.38 |
|  | IHD/CeVD | 6,472  (53) | Moderate | 0.27 |
|  |  |  | Severe | 0.68 |

Gender-stratified associations of interaction terms between depression severity and INFLA-score are reported. Final sample size (N) indicates the number of samples actually analyzed with a case-complete approach, after removing samples with missing covariates and/or event and follow-up data. Abbreviations: CVD = cardiovascular disease; IHD = ischemic heart disease; CeVD = Cerebrovascular disease.

**Table S5.**  Additive effect of INFLA-score on top of depressive symptom factors, on incident risk of **a)** hospitalizations and **b)** mortality, for all and specific causes.

**a)**

| **Cause of hospitalization** | **Polychoric factor** | **HR [95% CI]**  **(p-value)** |
| --- | --- | --- |
| **All causes** | **MR1+MR2** | **1.01[1.01; 1.02]**  **(<.0001)** |
| **CVD** | **MR1+MR2** | **1.02[1.01; 1.03]**  **(<.0001)** |
| **IHD** | **MR1+MR2** | **1.03[1.01; 1.05]**  **(0.0002)** |
| **CeVD** | **MR1+MR2** | **1.04[1.01; 1.06]**  **(0.001)** |

**b)**

| **Cause of death** | **Polychoric factor** | **HR [95% CI]**  **(p-value)** |
| --- | --- | --- |
| **All causes** | **MR1+MR2** | **1.03[1.01; 1.04]**  **(0.0003)** |
| **CVD** | **MR1+MR2** | **1.06[1.02; 1.09]**  **(0.0003)** |
| **IHD/CeVD** | **MR1+MR2** | **1.06[1.02; 1.10]**  **(0.002)** |

We report Hazard Ratios (HR) of hospitalizations and deaths for all and specific causes, with 95% Confidence Interval (CI) and relevant p-values, associated with a unit increase of INFLA-score, in multivariable Cox PH regressions (Model 2) including polychoric factors tagging somatic (MR1) and cognitive (MR2) symptoms. Significant HRs (p < 0.05) are highlighted in bold. Abbreviations: CVD = cardiovascular disease; IHD = ischemic heart disease; CeVD = Cerebrovascular disease.

**Table S6.** Interactive effects of depressive symptom factors and INFLA-score on incident risk of hospitalizations and mortality, for all and specific causes.

| **Event** | **Cause** | **N (events)** | **Polychoric factor**  **(interaction with INFLA-score)** | **p-value** |
| --- | --- | --- | --- | --- |
| Hospitalizations | All-cause | 6,689  (2,239) | MR1 | 0.56 |
|  |  |  | MR2 | 0.44 |
|  | CVD | 6,689  (630) | MR1 | 0.69 |
|  |  |  | MR2 | 0.43 |
|  | IHD | 6,689  (101) | MR1 | 0.34 |
|  |  |  | MR2 | 0.69 |
|  | CeVD | 6,689  (88) | MR1 | 0.42 |
|  |  |  | MR2 | 0.56 |
| Deaths | All-cause | 6,685  (156) | MR1 | 0.40 |
|  |  |  | MR2 | 0.19 |
|  | CVD | 6,683  (48) | MR1 | 0.44 |
|  |  |  | MR2 | 0.19 |
|  | IHD/CeVD | 6,683  (26) | MR1 | 0.84 |
|  |  |  | MR2 | 0.40 |

Association p-values of incident risk of hospitalization/deaths with interaction terms between polychoric factors tagging somatic (MR1)/cognitive symptoms (MR2) and INFLA-score are reported (but no HR estimates, as per PROC PHREG output). Final sample size (N) indicates the number of samples actually analyzed with a case-complete approach, after removing samples with missing covariates and/or event and follow-up data. Abbreviations: CVD = cardiovascular disease; IHD = ischemic heart disease; CeVD = Cerebrovascular disease.

**Table S7.** Gender-stratified analysis of the explanatory role of INFLA-score in the associations between depression severity, **a)** hospitalizations and **b)** mortality for all and specific causes.

**a)**

| **Cause of hospitalization** | **Gender** | **PTE (p-value)** |
| --- | --- | --- |
| All-cause | W | 1.5%  (0.07) |
|  | **M** | **3.0%**  **(0.02)** |
| CVD | W | 1.0%  (0.13) |
|  | **M** | **5.5%**  **(0.007)** |
| IHD | W | 1.8%  (0.12) |
|  | **M** | **11.0%**  **(0.01)** |
| CeVD | W | <1%  (NS) |
|  | **M** | **8.7%**  **(0.006)** |

**b)**

| **Cause of death** | **Gender stratum** | **PTE (p-value)** |
| --- | --- | --- |
| All-cause | W | 1.4%  0.18) |
|  | **M** | **20.5%**  **(0.01)** |
| CVD | W | <1%  (NS) |
|  | **M** | **24.5%**  **(0.01)** |
| IHD/CeVD | W | <1%  (NS) |
|  | M | <1%  (NS) |

Proportion of total effect explained (PTE) and relevant p-values as produced by the %MEDIATE macro are reported for each inflammation biomarker, in multivariable Cox PH models (Model 2). Significant PTEs (p < 0.05) are highlighted in bold. Abbreviations: CVD = cardiovascular disease; IHD = ischemic heart disease; CeVD = Cerebrovascular disease; W = women; M = men.

**Table S8.** Analysis of the explanatory role of INFLA-score in the associations between depressive symptoms factors, **a)** hospitalizations and **b)** mortality for all and specific causes.

**a)**

| Cause of hospitalization | Polychoric factor | PTE (p-value) |
| --- | --- | --- |
| **All-cause** | **MR1** | **2.5%**  **(0.006)** |
|  | MR2 | 1.7%  (0.27) |
| **CVD** | **MR1** | **4.1%**  **(0.02)** |
|  | MR2 | 9.2%  (0.28) |
| **IHD** | **MR1** | **8.6%**  **(0.03)** |
|  | MR2 | <1%  (NS) |
| CeVD | MR1 | 13.0%  (0.07) |
|  | MR2 | <1%  (NS) |

**b)**

| **Cause of death** | **Polychoric factor** | **PTE (p-value)** |
| --- | --- | --- |
| All-cause | MR1 | 25.7%  (0.14) |
|  | MR2 | 2.1%  (0.32) |
| CVD | MR1 | 6.3%  (0.31) |
|  | MR2 | 25.9%  (0.33) |
| IHD/CeVD | MR1 | 7.0%  (0.23) |
|  | MR2 | <1%  (NS) |

Proportion of total effect explained (PTE) and relevant p-values as produced by the %MEDIATE macro are reported for each inflammation biomarker, in multivariable Cox PH models (Model 2). Significant PTEs (p < 0.05) are highlighted in bold. Abbreviations: CVD = cardiovascular disease; IHD = ischemic heart disease; CeVD = Cerebrovascular disease; MR1 = somatic symptoms factore; MR2 = cognitive symptoms factor.

**Moli-sani Study Investigators**

The enrolment phase of the Moli-sani Study was conducted at the Research Laboratories of the Catholic University in Campobasso (Italy), the follow up of the Moli-sani cohort is being conducted at the Department of Epidemiology and Prevention of the IRCCS Neuromed, Pozzilli, Italy.

**Steering Committee:** Licia Iacoviello*°(Chairperson), Giovanni de Gaetano* and Maria Benedetta Donati*.

**Scientific Secretariat:** Marialaura Bonaccio*, Americo Bonanni*, Chiara Cerletti*, Simona Costanzo*, Amalia De Curtis*, Augusto Di Castelnuovo^§^, Alessandro Gialluisi*°, Francesco Gianfagna°^§^, Mariarosaria Persichillo*, Teresa Di Prospero* (Secretary).

**Safety and Ethical Committee:** Jos Vermylen (Catholic University, Leuven, Belgio) (Chairperson), Renzo Pegoraro (Pontificia Accademia per la Vita, Roma, Italy), Antonio Spagnolo (Catholic University, Roma, Italy).

**External Event Adjudicating Committee**: Deodato Assanelli (Brescia, Italy), Livia Rago (Campobasso, Italy).

**Baseline and Follow-up Data Management:** Simona Costanzo* (Coordinator), Marco Olivieri (Campobasso, Italy), Teresa Panzera*.

**Data Analysis:** Augusto Di Castelnuovo^§^ (Coordinator), Marialaura Bonaccio*, Simona Costanzo*, Simona Esposito*, Alessandro Gialluisi*°, Francesco Gianfagna°^§^, Sabatino Orlandi*, Emilia Ruggiero*, Alfonsina Tirozzi*.

**Biobank, Molecular and Genetic Laboratory:** Amalia De Curtis* (Coordinator), Sara Magnacca^§^, Fabrizia Noro*, Alfonsina Tirozzi*.

**Recruitment Staff:** Mariarosaria Persichillo* (Coordinator), Francesca Bracone*, Teresa Panzera*.

**Communication and Press Office:** Americo Bonanni*.

**Regional Institutions:** Direzione Generale per la Salute - Regione Molise; Azienda Sanitaria Regionale del Molise (ASReM, Italy); Agenzia Regionale per la Protezione Ambientale del Molise (ARPA Molise, Italy); Molise Dati Spa (Campobasso, Italy); Offices of vital statistics of the Molise region.

**Hospitals:** Presidi Ospedalieri ASReM: Ospedale A. Cardarelli – Campobasso, Ospedale F. Veneziale – Isernia, Ospedale San Timoteo - Termoli (CB), Ospedale Ss. Rosario - Venafro (IS), Ospedale Vietri – Larino (CB), Ospedale San Francesco Caracciolo - Agnone (IS); Casa di Cura Villa Maria - Campobasso; Ospedale Gemelli Molise - Campobasso; IRCCS Neuromed - Pozzilli (IS).

***Department of Epidemiology and Prevention, IRCCS Neuromed, Pozzilli, Italy

°Department of Medicine and Surgery, University of Insubria, Varese, Italy

^§^Mediterranea Cardiocentro, Napoli, Italy

*Moli-sani Study Past Investigators are available at* [*https://www.moli-sani.org/?page_id=173*](https://www.moli-sani.org/?page_id=173)
